# Real-Time, Risk-Based Clinical Trial Quality Management in China: Development of a Digital Monitoring Platform

**DOI:** 10.2196/64114

**Published:** 2025-04-25

**Authors:** Min Jiang, Shuhua Zhao, Yun Mei, Zhiying Fu, Yannan Yuan, Jie Ai, Yuan Sheng, Ying Gong, Jingjing Chen

**Affiliations:** 1 State Key Laboratory of Holistic Integrative Management of Gastrointestinal Cancers, Beijing Key Laboratory of Carcinogenesis and Translational Research, National Drug Clinical Trial Center, Peking University Cancer Hospital & Institute, 52 Fucheng Rd, Haidian District, Beijing, China, 86 01088196664; 2 Key Laboratory of Carcinogenesis and Translational Research (Ministry of Education/Beijing), National Drug Clinical Trial Center, Peking University Cancer Hospital & Institute, Beijing, China; 3 Yidu Cloud (Beijing) Technology Co, Ltd, Beijing, China; 4 Pfizer (China) Research and Development Co, Ltd, Shanghai, China

**Keywords:** clinical trial, quality control, risk-based quality management, digital, natural language processing

## Abstract

**Background:**

With the improvement of the drug evaluation system in China, an increasing number of clinical trials have been launched in Chinese hospitals. However, traditional clinical trial quality management models largely rely on human monitoring and counting, which can be time-consuming and are likely to generate errors and biases. There is an urgent need to upgrade and improve the efficiency and accuracy of clinical trial quality monitoring systems in hospital-based research institutions within China.

**Objective:**

The objective of this study was to develop a digital monitoring platform that allows for the real-time monitoring and detection of risk points and provides warnings about risk points throughout the entire life cycle of clinical trials, on the basis of historical clinical trial quality control (QC) findings.

**Methods:**

Leveraging the risk-based quality management mindset, we built a digital dynamic monitoring platform by using big data analysis and automatic quantitative technology. Data from clinical trial QC reports generated during 2019 to 2023 in Beijing University Cancer Hospital, China, were used to train the automated classification tool, establish warning thresholds, and validate threshold values. Quality findings from the early-stage, interim-stage, and conclusion-stage QC rounds of clinical trials were rated by using 3 severity grades (minor, major, or critical) and classified into 5 categories (with 4 taxonomy levels under each category). QC report text was processed by using an automated natural language processing tool. All QC reports were grouped into 2 clusters via hierarchical clustering analysis. QC findings from the relatively high-risk cluster (reports that were more likely to have major and critical findings, as determined by experienced QC analysts) were used to determine warning threshold values for the monitoring platform (ie, the lowest number of findings was set as the threshold value for each specific study stage, Level-3 taxonomy, and severity grade combination).

**Results:**

The most frequently reported Level-3 taxonomies in QC reports from 2019 to 2022 were “Standard Procedure and Process,” “Safety Reporting,” and “Source Data Collection and/or Recording.” In total, 189 warning threshold values were established based on data from 1380 QC reports generated during 2019 to 2022, covering 3 severity grades, 21 Level-3 taxonomies, and 3 QC rounds. The warning thresholds were applied to 211 QC reports generated in 2023, of which 19.9% (n=42) triggered warnings. Similar patterns of QC findings, including the most frequently noted Level-3 QC findings, were observed between reports generated in 2023 and those from 2019 to 2022.

**Conclusions:**

In clinical practice, our tool would enable the automated monitoring and detection of risk points throughout all clinical trial stages; accurately identify the most relevant trial procedure and function line; and notify quality management personnel, in real time, to take prompt actions and dynamically prevent the recurrence of quality issues.

## Introduction

Over the past 20 years, with the improvement of the drug evaluation system and standards in China, an increasing number of domestic and cross-national multicenter clinical trials have been launched in Chinese hospitals. However, in many hospital-based clinical trial institutions, traditional quality management models largely rely on human monitoring and counting, which not only require a lot of time and resources but also likely result in errors and biases. Therefore, such models may not be able to identify risk points and trends in a timely manner or prevent the recurrence of quality issues. In the face of the increasing number and complexity of clinical trials, the optimization of quality management models has become an urgent need for clinical trial institutions in Chinese hospitals.

Risk-based quality management (RBQM)—recommended in guidelines issued by the US Food and Drug Administration [1] and European Medicines Agency [2] in 2013, as well as in the International Council for Harmonisation of Technical Requirements for Pharmaceuticals for Human Use (ICH) guidelines for good clinical practice in 2016 [3]—is a powerful approach that has been explored and applied in a growing number of trials and institutions [4,5]. In 2020, the China National Medical Products Administration introduced the concept of RBQM into Chinese trial quality management systems [6]. In 2022, the China Center for Drug Evaluation released Statistical Principles for Centralized Monitoring of Drug Clinical Trials, which was considered a milestone in China’s clinical trial revolution and a guideline for the improvement and implementation of RBQM in China’s clinical practice [7].

To meet the urgent need for upgrading quality management models and achieving better efficiency, accuracy, and timeliness, we leveraged the RBQM mindset and used a long-term collection of quality management data from clinical trial programs in Beijing University Cancer Hospital, China, to build a digital dynamic monitoring platform via big data analysis and automatic quantitative technology. Through this platform, we aim to dynamically and prospectively monitor the entire life cycle of clinical trials and efficiently identify, intervene with, and systematically prevent quality risks that might compromise critical trial processes, patient safety, or data integrity.

## Methods

### Ethical Considerations

This study was based on the processing and analysis of quality control (QC) documents from a large number of clinical trials, which underwent their own ethics reviews. Further, the text of the QC findings did not contain personal information of trial participants. As such, this study did not require ethics board review.

### Materials

A total of 1591 QC reports were generated in our institution from January 2019 to September 2023, involving 993 clinical trials. In accordance with relevant quality management standard operating procedures, a specific QC group was responsible for conducting at least 3 rounds of routine QC for each trial, including but not limited to (1) an early-stage QC round at the time point of achieving 5 enrollments or at 3 months after trial initiation, (2) an interim-stage QC round once every 6 months or when the trial achieved 20 enrollments, and (3) a conclusion-stage QC round at the time of site closeout. A detailed QC report was generated for each QC round, recording all quality issues. To build a digital monitoring platform, 1380 reports, which were generated during 2019 to 2022, served as the foundation for automated quantitative processing and the identification of warning thresholds, while the remaining 211 reports, which were generated in the year 2023, were used for testing the effectiveness of those thresholds in identifying high-risk trials.

### Classification of QC Findings

Findings from the routine QC reports were rated by using 3 severity grades (minor, major, or critical) and classified into 5 categories (“Data,” “Quality,” “Safety and Ethics,” “Equipment and Facility,” or “Finance”), with 4 taxonomy levels under each category. The severity ratings and Level-1 to Level-3 taxonomies would allow for an overall review and trend analysis, while the Level-4 taxonomies were designed to trace back precisely to the corresponding clinical trial procedure and personnel.

### Novel Automated Classification Tool

**Figure 1.**
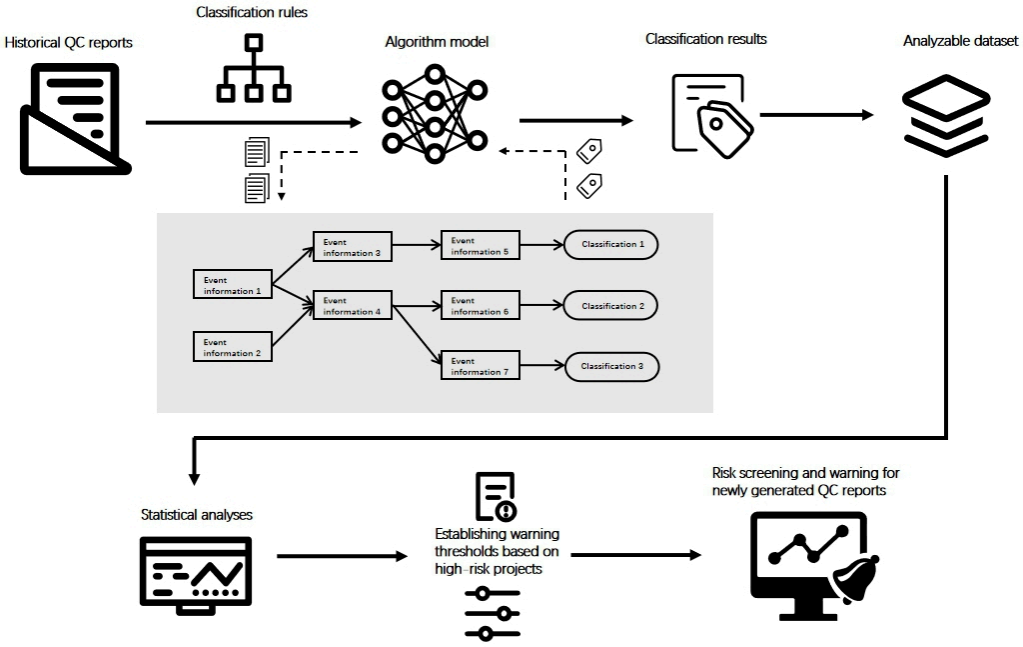
Algorithm classification tool flowchart. QC: quality control.

To enable the automated classification of QC findings, we developed a natural language processing (NLP) algorithm based on the Local Interpretable Model-Agnostic Explanations framework, which was implemented in Python. This algorithm was designed to perform multidimensional classifications of events in QC report texts (Figure 1).

The dataset used to develop the NLP algorithm comprised 1380 QC reports that were generated during 2019 to 2022; an average of 345 reports were generated per year. Among QC findings from these reports, 55.48% were categorized as minor findings, 42.23% were categorized as major findings, and 2.29% were categorized as critical findings. The word count of the reports ranged from 1619 to 17,367 words, with a median word count of 5938 (IQR 4453-7422) words per report. Data were stratified and split into training, validation, and testing sets (70%, 15%, and 15%, respectively) to ensure balanced class representation. Annotation rules covered 4 entity dimensions and 54 issue categories, which were derived from industry standards and refined through discussions with an internal expert team.

In the first phase, the dataset was annotated by using multidimensional information labeling rules, generating high-quality training data. In the second phase, natural text from QC reports was subjected to preliminary automated classification via a Bayesian network, which determined the Level-1 to Level-3 classifications for each text segment based on joint probability. Additionally, semantic role labeling—a critical step in the classification NLP tool—was used to identify the predicate-argument structure of each sentence [8]. After training with the dataset, an explainable directed acyclic graph was constructed [9]. In the directed acyclic graph, graph neural networks were used to propagate information across the graph, forming an algorithmic model capable of capturing events. The model achieved precision (P) and recall (R) scores >0.95. In the third phase, by using the trained model, events were extracted from the QC report text and subjected to Bayesian inference. If an inference could be drawn from the graph, automatic classification was completed; if no inference could be made, a new branch was created, and the entire graph model was updated and iterated. The final Level-4 classification results were represented as the terminal leaf nodes in the graph.

### Clustering

After all findings from QC reports were classified by using our automated classification tool, a dataset was created based on the incidence rates of different categories of QC findings from each QC report. Using SPSS for Windows 20.0 (IBM Corp), hierarchical clustering analysis was applied to this dataset to ensure that reports with similar patterns of QC findings were grouped into the same cluster: each report was initially treated as a separate cluster; then, the algorithm identified 2 clusters that were closest to each other (ie, clusters with maximum similarity) and merged these 2 clusters into 1 new cluster. These steps were repeated until all QC reports were grouped into 2 clusters (Cluster I and Cluster II). A dendrogram was generated, showing the levels of similarity and dissimilarity between QC reports. Per manual interpretation by a group of experienced QC analysts, Cluster II was identified as high risk (ie, reports with more findings or a higher proportion of major and critical findings), and Cluster I was considered relatively “normal” (reports with less findings or a higher proportion of minor findings).

### Establishment of Warning Thresholds and Detection of Risk Points

Warning thresholds were established based on the high-risk (Cluster II) QC reports from 2019 to 2022; the lowest number of findings was set as the threshold value for a specific study stage, Level-3 taxonomy, and severity grade combination. For example, a threshold value of X was set for the following combination: early-stage QC round, Level-3 taxonomy “Biological Sample Management,” and major findings; this made sure that all high-risk QC reports from early-stage QC rounds had at least X major findings under the Level-3 taxonomy “Biological Sample Management.” The warning thresholds were then applied to QC reports from 2023; these warning thresholds would help with determining the risk points (ie, the most commonly reported findings) and investigating the root causes of findings.

## Results

### Summary of 2019 to 2022 QC Findings

**Figure 2.**
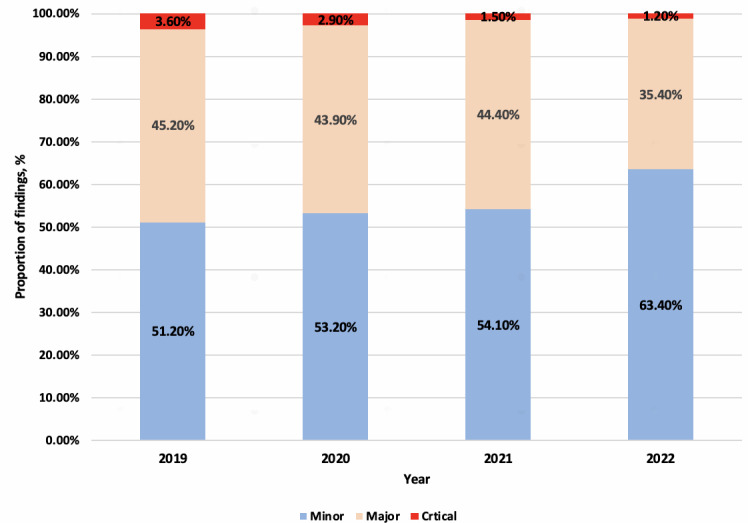
Proportion of quality control findings under 3 categories of clinical significance and severity.

From 2019 to 2022, the proportion of major or critical findings generally decreased each year (major findings showed a 1.27-fold decrease, and critical findings showed a 3-fold decrease), even during the COVID-19 pandemic, while the proportion of minor findings showed an approximately 1.24-fold increase over the 4 years (Figure 2).

**Figure 3.**
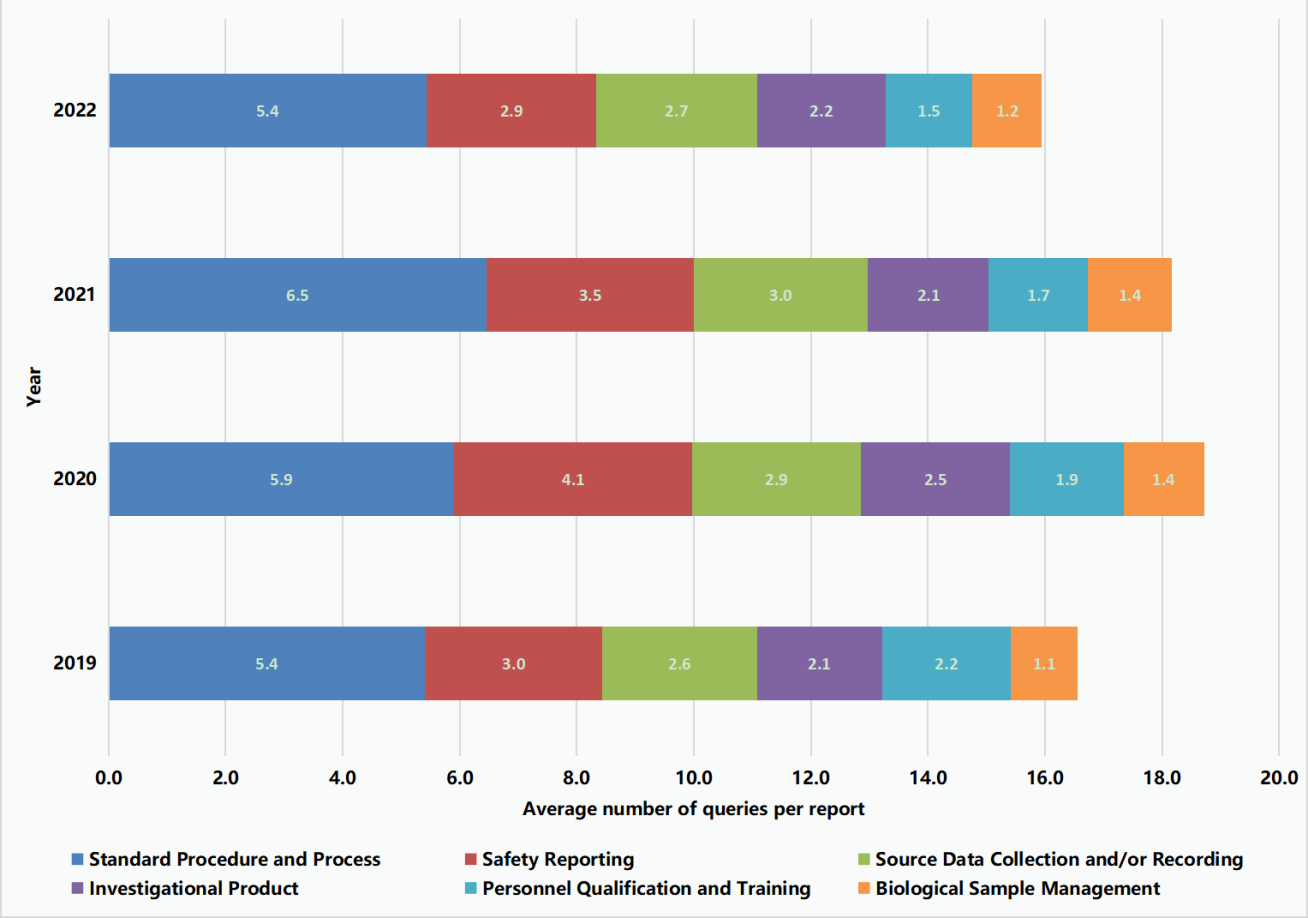
Average number of findings per quality control report under the most frequently reported Level-3 taxonomies (proportions of findings for these taxonomies: >5%).

It was consistently observed during each year that the most commonly reported Level-3 taxonomies (the proportions of findings for these taxonomies were >5%), in decreasing order of frequency, were (1) “Standard Procedure and Process,” (2) “Safety Reporting,” (3) “Source Data Collection and/or Recording,” (4) “Investigational Product,” (5) “Personnel Qualification and Training,” and (6) “Biological Sample Management” (Table 1). In 2020, under the influence of the COVID-19 pandemic, increases were observed in the average numbers of findings per report that fell under these Level-3 taxonomies, but 2 years later, the average numbers of these findings dropped back to their 2019 levels (Figure 3).

**Table 1.** Proportion of quality control (QC) findings under each Level-3 taxonomy from 2019 to 2023.

Level-3 classification	2019 (% of QC findings)	2020 (% of QC findings)	2021 (% of QC findings)	2022 (% of QC findings)	2023 (% of QC findings)
Source Data Collection and/or Recording	13.62	13.65	13.75	13.53	13.61
Source Data Modification/Correction	0.62	0.41	0.23	0.31	0.44
Source Data Transcription	0.74	0.72	0.56	0.70	0.73
Process Documentation Management	0.08	0.07	0.08	0.09	0.08
Standard Procedure and Process	26.47	27.14	27.95	26.91	26.97
Personnel Qualification and Training	9.21	8.02	7.81	9.02	9.09
Query Identification	0	0	0	0	0
Query Recording and Reporting	0.51	0.27	0.36	0.27	0.58
Query Prevention	0	0	0	0	0
Laboratory, Equipment, Facility, and Supply Management	1.20	1.29	1.55	1.28	1.23
Biological Sample Management	5.58	5.72	6.35	5.88	5.59
Clinical Trial Document Management	3.98	4.21	4.35	4.07	4.09
Human Genetic Resource Management	0.23	0.19	0.17	0.24	0.24
Informed Consent Personnel Qualifications	1.40	1.22	1.59	1.50	1.48
Informed Consent Process	2.29	2.23	2.37	2.47	2.39
Ethics Committee Composition and Operation	1.17	1.70	1.34	1.53	1.23
Safety Reporting	16.50	16.97	15.79	15.94	15.98
Concomitant Medications	3.34	3.18	2.91	2.98	3.19
Investigational Product	11.52	11.43	11.50	11.76	11.57
Inclusion and Exclusion Criteria	1.53	1.58	1.34	1.52	1.51
Clinical Trial Finance Process	0	0	0	0	0

**Figure 4.**
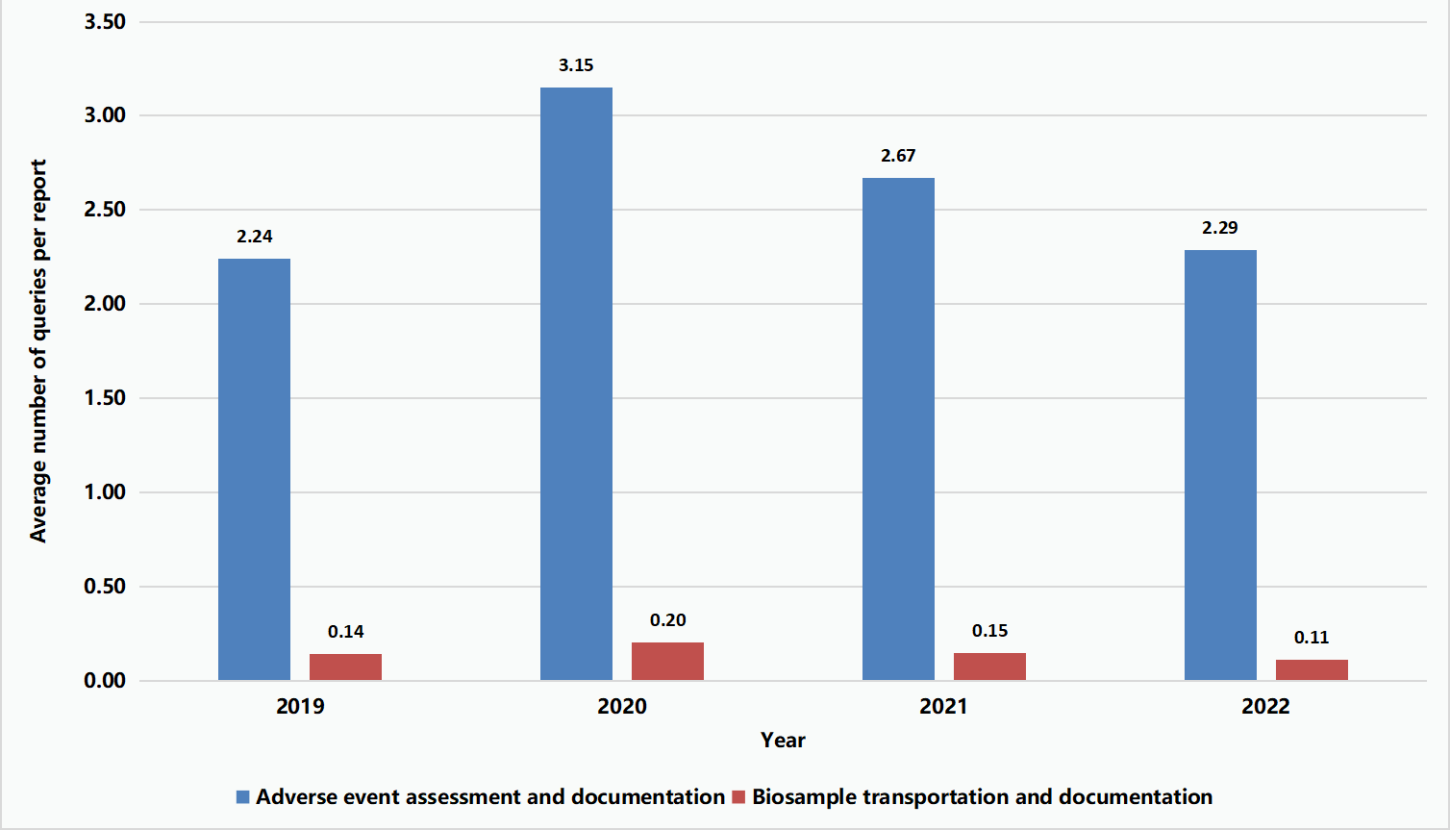
Trends of change in average number of findings per quality control report under two Level-4 taxonomies from 2019 to 2022.

Similar trends were also observed when QC findings were summarized under Level-4 taxonomies. A 1.41-fold increase in the average number of findings under the Level-4 taxonomy “Adverse Event Assessment and Documentation” was observed in 2020 when compared to 2019. Further, while the average number of findings under the Level-4 taxonomy “Biological Sample Shipment and Recording” was ≤0.2 per report across each year, a 1.43-fold increase was also noted in 2020 when compared to the previous year. These trends were likely attributable to the outbreak of the COVID-19 pandemic. With the adaptive actions taken in response to COVID-19 (eg, implementation of digital monitoring), the numbers of QC findings under these two Level-4 taxonomies eventually dropped back to their 2019 levels (Figure 4).

### Warning Thresholds and Warning-Triggering Risks in the Year 2023

A total of 189 warning threshold values were established to cover all 3 severity grades, 21 Level-3 taxonomies, and 3 rounds of QC (Multimedia Appendix 1). These thresholds were applied to a total of 211 QC reports generated in 2023. Among those reports, 42 triggered warnings, resulting in a trigger rate of 19.9%. Approximately half (n=23, 55%) of the 42 warning-triggering, high-risk reports were from interim-stage QC rounds, while 8 and 11 were from early-stage and conclusion-stage QC rounds, respectively.

**Figure 5.**
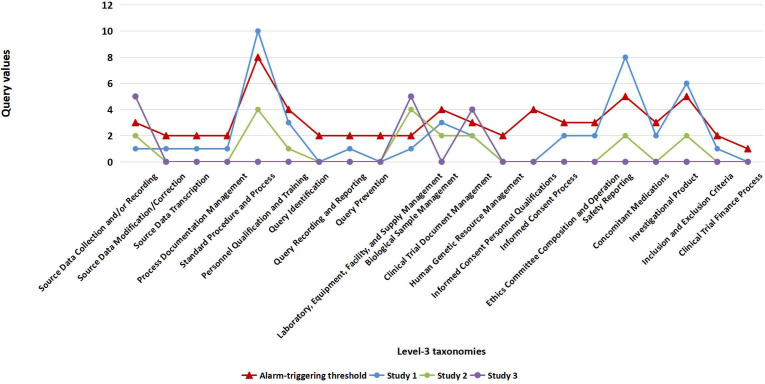
Number of major QC findings under each Level-3 taxonomy from 3 early-stage QC reports, in comparison with corresponding threshold values. QC: quality control.

Figure 5 presents data from 3 randomly selected reports from early-stage QC rounds as examples to show the number of major QC findings that exceeded or did not exceed the corresponding threshold value for each of the 21 Level-3 taxonomies.

Among the 42 high-risk reports in 2023, after the application of warning threshold values, the most frequently noted Level-3 taxonomies with alarm-triggering QC findings were generally consistent with those observed in QC reports from 2019 to 2022 (Tables1  2, Figure 3). The most common alarm-triggering Level-3 taxonomy was “Standard Procedure and Process,” which was reported in 36% (15/42) of high-risk reports, and its most relevant process (ie, “risk point,” as defined by Level-4 taxonomies) was “Protocol Deviation – Deviation from Study Design,” which was reported in 33% (5/15) of the reports that triggered an alarm for QC findings that fell under the Level-3 “Standard Procedure and Process” taxonomy. In response to Level-4 taxonomies that were more likely to cause alarm-triggering QC issues (Table 2), intervention strategies to be incorporated into routine clinical practice were proposed.

**Table 2.** Most frequently reported Level-3 taxonomies in alarm-triggering quality control reports from 2023 (n=42) and their corresponding most relevant Level-4 taxonomies.

Level-3 taxonomy	Level-3 taxonomy incidence, number of reports (%)	Most relevant Level-4 taxonomy
Standard Procedure and Process	15 (36)	Protocol Deviation – Deviation From Study Design
Safety Reporting	8 (19)	Adverse Event Assessment and Documentation
Source Data Collection and/or Recording	8 (19)	Paper Source Data Files
Biological Sample Management	6 (14)	Biosample Transportation and Documentation
Personnel Qualification and Training	6 (14)	Personnel Authorization and Documentation
Investigational Product	6 (14)	Administration of Investigational Products and Associated Documentation Issues
Laboratory, Equipment, Facility, and Supply Management	4 (10)	Equipment Maintenance Issues
Clinical Trial Document Management	4 (10)	Clinical Trial Document Maintenance
Concomitant Medications	4 (10)	Concomitant Medications Record and Documentation
Informed Consent Process	3 (7)	Informed Consent Signing Issues
Process Documentation Management	3 (7)	Quality Control Documentation
Inclusion and Exclusion Criteria	2 (5)	Inclusion and Exclusion Criteria Documentation
Query Recording and Reporting	1 (2)	Incomplete Monitoring Records

## Discussion

As stated in the ICH E6(R3) guideline published in January 2025, clinical trial institutions and sponsors are encouraged to use innovative digital technologies to improve clinical trial quality, taking into consideration the risk factors that could potentially impact participant safety, data quality, and other key elements in clinical trial procedures [3]. In this study, data from historical clinical trial QC reports generated in our hospital were standardized and classified for further risk-based qualitative analyses, and the minimum number of QC findings was determined as the threshold value for each QC stage, severity level, and Level-3 taxonomy combination. These QC warning thresholds were embedded into our digital dynamic monitoring platform to allow for the dynamic and proactive identification of high-risk QC reports and to notify quality management personnel, in a real-time manner, about which reports had a number of QC findings (ie, in a specific stage, severity level, and Level-3 taxonomy) that was larger than the corresponding threshold value. For example, a QC warning will be triggered for an early-stage QC report if it reports more than 13 minor findings, more than 3 major findings, or more than 2 critical findings under the Level-3 taxonomy “Source Data Collection and/or Recording,” requesting the quality management group to follow up and take actions.

With the help of the digital platform, the quality management team can quickly and accurately identify the relevant clinical trial procedure and function line for the reported quality issues and adopt mitigation strategies. For example, in QC reports from 2019 to 2022, “Standard Procedure and Process” was the most frequently reported Level-3 taxonomy for QC reports generated during the conclusion stage (ie, around the time of site closeout), and one-third (38/114, 33.3%) of the quality issues were attributed to participants’ poor compliance with safety visits after they left the trial site. Driven by this observation, we learned from participants that many of them lived far away from our trial site in Beijing, and after leaving the trial site, it was difficult for them to travel a long distance back to the trial site for additional safety follow-ups. Our proposed action is to allow participants who are discharged from the trial site to complete their follow-up visits in a local clinical trial center (ie, at or near their hometown) equipped with a remote visit system, as an approach to improving their compliance with the safety monitoring procedures. Another example is the quality alarm associated with the Level-4 taxonomy “Administration of Investigational Products and Associated Documentation Issues.” In a trial with QC findings that fell under this taxonomy, the study drug was to be orally self-administered at home, and a large proportion of participants missed multiple oral doses. In response to this finding, our proposed action is to send drug administration reminders via participants’ social networking platforms and the hospital’s mobile app. The digital platform also enables the study team to conduct targeted source data verifications and reviews; the study team can also exempt a trial from complete source data verifications and reviews if it did not trigger any warnings.

The digital platform remains to be optimized for future clinical practice; the input of more QC data and an upgraded automated calculation tool are needed to enhance its accuracy and applicability. However, the concurrent use of other digital tools may further enhance the efficiency of quality management systems. For high-risk trials that have already triggered an alarm in the early- or interim-stage QC round, a remote monitoring system may help to reduce quality issues due to trial procedure noncompliance.

## Supplementary material

10.2196/64114 Multimedia Appendix 1 Threshold values for Level-3 taxonomies in quality control (QC) across 3 study stages and 3 severity grades.

## Abbreviations

ICH: International Council for Harmonisation of Technical Requirements for Pharmaceuticals for Human Use
NLP: natural language processing
QC: quality control
RBQM: risk-based quality management
