# Supplementary material for: Real-Time, Risk-Based Clinical Trial Quality Management in China: Development of a Digital Monitoring Platform
Source: JMIR Med Inform. 2025 Apr 25;13:e64114. doi: 10.2196/64114 (PMC12047851; doi:10.2196/64114)
Supplement: Multimedia Appendix 1 [file medinform-v13-e64114-s001.docx]

**Multimedia Appendix 1. Threshold values for Level-3 taxonomies in quality control (QC) across 3 study stages and 3 severity grades**

|  | **Early-Stage QC** | | | **Interim-Stage QC** | | | **Conclusion-Stage QC** | | |
| --- | --- | --- | --- | --- | --- | --- | --- | --- | --- |
| **Level-3 Taxonomy** | **Minor** | **Major** | **Critical** | **Minor** | **Major** | **Critical** | **Minor** | **Major** | **Critical** |
| Source Data Collection and/or Recording | 10 | 3 | 2 | 16 | 5 | 2 | 12 | 4 | 2 |
| Source Data Modification/Correction | 3 | 2 | 1 | 4 | 2 | 1 | 4 | 2 | 1 |
| Source Data Transcription | 3 | 2 | 1 | 4 | 3 | 2 | 4 | 3 | 2 |
| Process Documentation Management | 3 | 2 | 1 | 4 | 2 | 1 | 4 | 2 | 1 |
| Standard Procedure and Process | 10 | 8 | 2 | 18 | 15 | 3 | 16 | 12 | 2 |
| Personnel Qualification and Training | 8 | 4 | 2 | 10 | 6 | 2 | 9 | 5 | 2 |
| Query Identification | 3 | 2 | 1 | 4 | 2 | 1 | 4 | 2 | 1 |
| Query Recording and Reporting | 3 | 2 | 1 | 4 | 2 | 1 | 4 | 2 | 1 |
| Query Prevention | 3 | 2 | 1 | 4 | 2 | 1 | 4 | 2 | 1 |
| Laboratory, Equipment, Facility, and Supply Management | 4 | 2 | 2 | 5 | 3 | 2 | 4 | 2 | 2 |
| Biological Sample Management | 5 | 4 | 2 | 8 | 6 | 2 | 7 | 5 | 2 |
| Clinical Trial Document Management | 6 | 3 | 2 | 8 | 3 | 2 | 7 | 2 | 2 |
| Human Genetic Resource Management | 3 | 2 | 2 | 5 | 3 | 2 | 4 | 2 | 2 |
| Informed Consent Personnel Qualifications | 5 | 4 | 2 | 5 | 4 | 2 | 5 | 4 | 2 |
| Informed Consent Process | 4 | 3 | 2 | 5 | 4 | 2 | 4 | 3 | 2 |
| Ethics Committee Composition and Operation | 4 | 3 | 2 | 5 | 4 | 2 | 4 | 3 | 2 |
| Safety Reporting | 8 | 5 | 2 | 14 | 10 | 2 | 12 | 8 | 2 |
| Concomitant Medications | 6 | 3 | 2 | 8 | 7 | 2 | 7 | 6 | 2 |
| Investigational Product | 6 | 5 | 2 | 12 | 10 | 3 | 10 | 7 | 3 |
| Inclusion and Exclusion Criteria | 3 | 2 | 2 | 4 | 3 | 3 | 4 | 3 | 3 |
| Clinical Trial Finance Process | 2 | 1 | 1 | 2 | 1 | 1 | 2 | 1 | 1 |
